# Do natural catastrophic events and exceptional climatic conditions also affect parasites?

**DOI:** 10.1017/S0031182023000471

**Published:** 2023-10

**Authors:** Giovanni Poglayen, Antonio Gelati, Antonio Scala, Salvatore Naitana, Vincenzo Musella, Martina Nocerino, Giuseppe Cringoli, Antonio Frangipane di Regalbono, Annette Habluetzel

**Affiliations:** 1 Department of Veterinary Medical Science, Alma Mater Studiorum University of Bologna, Bologna, Italy; 2 Department of Veterinary Medicine, University of Sassari, Sassari, Italy; 3 Department of Health Sciences, University of Catanzaro Magna Græcia, Catanzaro, Italy; 4 Department of Veterinary Medicine and Animal Production, University of Naples Federico II, Naples, Italy; 5 Department of Animal Medicine, Production and Health, University of Padova, Padova, Italy; 6 School of Pharmacy, University of Camerino, Camerino, Italy

**Keywords:** Drought, fire, flood, natural disasters, parasites

## Abstract

Parasites and parasitologists have always lived together in good and bad luck in a sort of forced marriage. In recent times bad luck certainly prevailed, because of increasing man-made emergencies such as wars, chemical disasters, but also because of natural disasters, amplified by climate change, that condition more and more parasite–host equilibrium. The symposium at the National Congress of the Italian Society for Parasitology, was a first occasion for Italian parasitologists to reason about ‘disaster parasitology’ and researchers’ responsibilities. Extreme weather events and their impacts on parasites’ epidemiology are illustrated, comparing disasters that recently occurred in Italy with literature data. In particular, the Sardinian Island was hit subsequently by fires and floods exacerbating the effects on ecosystems and parasite–host-relationships. Examples of *Cryptosporidium* outbreaks in man and *Fasciola hepatica* infections in various hosts after heavy rains are reviewed and effects of droughts on pasture borne parasites, such as gastro-intestinal nematodes of ruminants are discussed. Heavy rains may also cause dissemination of toxic substances released accidentally from chemical plants as happened e.g. in Milan province (IT) after the dioxin hazard. The overlapping effects of strictly man-made disasters with climate change dependent extreme weather events is further challenging the understanding of what are the consequences of disasters on ecosystems and parasite epidemiology.

GIS applications combined with AI programs may help to face the complex challenges, allowing the collection and analysis of spatial/temporal data at whatever level desired. Examples illustrated in the article suggest their employment also in a more systematic, prevention-oriented manner.

## Graphical abstract

**Figure:**
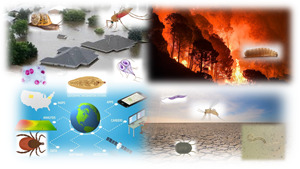

## Introduction

Parasites and parasitologists have always lived together in good and bad luck in a sort of forced marriage. In recent times bad luck certainly prevailed, because of increasing man-made emergencies such as wars and civil conflicts, chemical disaster, but also because of natural disasters, amplified by climate change, that condition more and more parasite–host equilibria.

Since a few decades, global warming – a phenomenon undisputedly linked to human activities – has become a major concern and a subject of research among parasitologists. The rise of mean temperatures is expected to speed up the development of environmental parasite stages such as oocysts, eggs and larvae, increase the abundance of poikilothermic intermediate hosts (e.g. insects, gastropods) and accelerate parasite maturation in them. However, the expected consequent increase in transmission intensity might be tempered by decreased survival rates of the same parasite stages and intermediate hosts at higher temperatures. Comparatively, limited attention is being paid to the effects of extreme weather events on parasite diffusion and infection risks, although catastrophic events such as floodings, droughts and fires are increasing in frequency and intensity. Nearly two-thirds of European human and domestic animal pathogens are estimated to be climate sensitive often to more than one climate driver (e.g. temperature and rainfall and humidity) (McIntyre *et al*., 2017). In the case of arthropod intermediate hosts, the development and replication of pathogens within the vectors is favoured by increasing temperature, as it is, the development and survival of the vectors themselves (Caminade *et al*., 2019). On the top, there are various socio-economic factors which contribute to the emergence and spread of parasitic and infectious diseases: (i) population growth and the contemporaneous increase of animal populations, are increasing contacts between humans and animals in both domestic and wild environments; (ii) conflicts and political instability cause forced displacements of people and animals that determine precarious sanitary conditions and increase the recrudescence of various parasitic and infectious diseases.

In the Italian context, in many of the disasters that hit the country during the last decades, the role of veterinarians remained unclear and only following more recent important telluric events their importance as professionals was officially recognized and defined by law (AA.VV., 2002). However, parasitologists whose scientific and cultural souls have always linked together medical, biological and veterinary disciplines, adhered always to the One Health approach, a concept finally fully recognized in the Italian health sector.

From literature and personal observations of the authors, shared during the National Congress of the Italian Society of Parasitology (SoIPa), held in Naples in June 2022, various examples emerged (with just a mention of 2 chemical emergencies that made history) suggesting that drastic impairment or complete disruption of parasite host equilibria may occur, leading to parasite elimination or epidemics. However, a light of hope for tackling present and future disaster related challenges, is represented by new, highly advanced and interactive technological and digital tools such as Geographic Information Systems (GIS) and electronic data collection devices such as surveillance drones.

## The Sardinia ‘laboratory’ – an Italian region particularly hit by exceptional climatic events

Also in the Mediterranean region, changes in environmental conditions caused by deforestation and fires, together with the more and more frequent occurrence of extreme climatic conditions such as extended periods of drought and/or intense rainfall followed by flooding, have become increasingly frequent. These led to significant alterations in grazing areas in terms of availability and quality of fodder present for herbivorous hosts, but also impact on microclimate suitability and consequently on parasite environmental habitats, their survival and capacity to propagate. It is obvious that extreme climatic episodes and the phenomena resulting from them, such as prolonged drought periods, accompanied by extremely high temperatures and particularly windy conditions, can lead to the burst of fires of vast proportions. Unfortunately, these events have been occurring more and more frequently in Sardinia, an island characterized by extensive livestock husbandry such as sheep farming, displaying territorial characteristics to a wide extend representative for the Mediterranean area. These events are often taking the livestock sector by surprise and cause considerable direct economic losses. Impacting on the animals’ health, disasters affect also host resilience to parasites, thus the epidemiology of parasitic diseases is foreseeable to undergo relevant changes influenced by many factors, which are difficult to quantify. Two examples of extreme environmental/climatic events that have recently occurred in Sardinia, may help to elucidate the issue: namely, the major flood in November 2013 that stroke the north-eastern part of the island (municipalities of Torpè and Lodè), and the massive fire that devastated in summer 2021 the Montiferru area (central-western area of Sardinia). Considerations made in this article are prevalently regarding the possible effects of these phenomena on the epidemiology of endoparasitoses of grazing animals.

Various effects may occur as a consequence of the ‘opposite’ phenomena above-mentioned: floods may lead to an increase in the spread of some parasitosis such as cryptosporidiosis, toxoplasmosis, giardiosis and fasciolosis, while in areas affected by fires a drastic reduction is expected of certain propagation forms (oocysts, L3 larvae of gastro-intestinal nematodes), intermediate hosts (oribatid mites, xerophilous gastropod mollusks), as well as ixodid tick vectors of piroplasmosis or pupae of myasis agents (e.g. *Hypoderma* spp., *Oestrus ovis*, *Gasterophilus* spp.). However, these 2 phenomena, one after the other, may potentiate the catastrophic effects: fires will cause soil hydrophobicity due to the mineralization of the organic substance of its surface layer and subsequent heavy rainfalls will then increase the risk of floodings. In fact, it has been observed in fire affected areas in Sardinia that the soil surface layer decreased its water absorption capacity causing an increase in the speed of waterflow with significant erosion phenomena and flood risks downstream (Camarda and Vacca, 2021).

## Floods: a concern for a wide range of parasites – but not only

### Impact on waterborne protozoan parasites

Both, floodings and droughts can lead to epidemics caused by waterborne protozoan parasites: cysts of *Giardia intestinalis,* oocysts of *Cryptosporidium parvum* and *Toxoplasma gondii*, due to their small size and specific weight close to that of water, may get flushed from soil surfaces, transported by run-off water to various types of water reservoirs and thus contaminate such water sources destinated for the provision of drinking water. On the other hand, drought conditions may force animals and humans to resort to unsafe water resources, infecting themselves with waterborne pathogenic agents (Cann *et al*., 2013). Whereas the latter situation may occur more frequently in countries with poor sanitary infrastructures, flood borne epidemics caused by protozoal agents, mainly *Cryptosporidium*, have been observed to a major extend in the northern hemisphere and reported from Europe and the United States (Karanis *et al*., 2007). A large outbreak caused by *Cryptosporidium* occurred in the city of Milwaukee in 1993 causing diarrhoeal disease in 403 000 inhabitants (MacKenzie *et al*., 1994). Inefficiency of drinking water treatment plants was found to be the likely cause. In fact, after strong thunderstorms the plants were challenged with highly turbid water overriding their capacity of maintaining adequate removal of pathogens by the coagulation and filtration process (Leland *et al*., 1993; Corso *et al*., 2003).

In the nineties, several *Cryptosporidium* outbreaks were reported in the UK. More than 500 cases of diarrhoeal disease were notified in Sheffield and in Swindon & Oxfordshire, respectively (Karanis *et al*., 2007) and about 1840 individuals were affected by the outbreak of Warrington. Interestingly in all the three events *Cryptosporidium* infections were of zoonotic origin. Flooding of pastures grazed by oocysts eliminating cattle, had led to the contamination of the drinking water. In the case of Warrington, an area geologically characterized by sandstone, the heavy rainfalls most likely have caused oocysts bypass of natural sandstone filtration. This example illustrates the importance of including geological assessments in any endeavours to estimate the risk of flood related epidemics.

Extreme heavy rains may also lead to the inundation of pastures with manure, industrial waste waters or raw sewage water. Thus, pig and beef farms not only constitute a potential source of human infections but extensively raised beef themselves risk of getting infected/contaminated when feeding on pastures polluted with microbial or chemical agents, protozoan parasite cysts or even with *Taenia saginata* eggs in case of inundation with sewage water of human origin (Crist *et al*. (2020).

Two succeeding large toxoplasmosis outbreaks stroke the city of Victoria (British Columbia, Canada) in October 1994 and April 1995, involving 2894 and 7718 inhabitants respectively. Also in this case, oocyst contamination of municipal drinking water was responsible for the epidemics. The events occurred following periods of excess rainfall that led to fecal contamination of the water reservoir by domestic and feral cats and cougars, found *Toxoplasma* positive in subsequent investigations (Karanis *et al*., 2007).

Not only terrestrial but also aquatic environments are at risk of increased pathogen contamination because of climate change. Investigating watersheds of coastal regions in California, USA, vanWormer *et al*. (2016) found highest *T. gondii* oocyst levels in regions of increased *T. gondii* infection monitored in sentinel marine mammals. Heavy precipitation phenomena raised oocyst delivery to the ocean by 79%. Thus, extreme climatic events combined with anthropogenic changes in landscapes can accelerate runoff of diverse pathogens from terrestrial to aquatic environments, increasing the risk of transmission to people, domestic animals and wildlife in coastal areas (vanWormer *et al*., 2016).

### Impact on soil-borne parasites and fluke diseases

In Haiti, deforestation, heavy rains and subsequent silting of a river, led to periodic flooding of the area of Leogane town with the deposition of a sandy loam topsoil increasing soil moisture (Lilley *et al*., 1997). A longitudinal coprological study, that followed up small children for about 6 years (1990–96), allowed to detect an impact of the environmental change on the local hookworm cycle. Following years of regular flooding events, the prevalence of hookworm infected children was found increased from zero in 1990 to about 15% in 1996, while other helminthic infections remained constant. Increased soil moisture, given by the sandy loam depositions after river floodings, might have provided an ideal environment for the larval development of hookworms and thus facilitated the infection of children with this parasite (Lilley *et al*., 1997).

Worth mentioning are 2 recent cases of human fasciolosis (HF) in Belgium, probably caused by eating metacercariae contaminated vegetables from a garden that was inundated by runoff from pasture after heavy rains. Because native HF is rare and the transmission route was unusual, HF was not diagnosed until 6 months after symptom onset in both cases (Milas *et al*., 2020).

Regarding fasciolosis disease in sheep farms in endemic areas of Italy, outbreaks were found to be associated to a significant increase (*P* < 0.001) of temperature and of the number of rainy days in those areas compared to previous years’ data (Bosco *et al*., 2015). Taking into consideration all the Mediterranean area, the potential effects of these phenomena on the epidemiology of *F. hepatica* and the implications for sheep farming have been illustrated in a recent overview by Alba *et al*. (2021).

When man-made disasters such as wars superimpose effects of extreme events caused by climate change, parasites may take profit of newly created habitats and facilitated transmission conditions. Once again war has brought about deleterious effects on human, animal and environmental health (Mantovani *et al*., 2006) during the events that devastated the Balkans from 1991 to 2001. Croatian Public Health Services revealed a dramatic increase in human trichinellosis cases in a country already considered to be at high risk of this infection (Marinculic, personal com.). If this was foreseeable due to the collapse of the public health service, the same cannot be said for what has been recorded in wildlife regarding a trematode parasite: *Fascioloides magna* in Europe is an imported liver fluke of game animals, such as red deer, not previously reported in Croatia. In non-specific hosts such as domestic ruminants, pigs, horses and rodents parasite infection is frequently fatal. In 1999 the parasite was found in deer of the Baranja region (Marinculic *et al*., 2002). Among the possible causes of its occurrence, Croatian parasitologists recall floods that may have transferred infective eggs and intermediate hosts from endemic areas of Hungary, but also mentioned, that the migration of deer, disturbed by war operations, to Hungary and their subsequent return to Croatia after the war, might explain the *F. magna* introduction in this country (Marinculic *et al*., 2002).

### Impact on fluke disease and risk of metacestadosis in Sardinia

Recently, in Ottana, (Nuoro province), after very heavy rains at the end of September 2020, acute fasciolosis cases in sheep farms were found to be increased, aggravated by a compromised nutritional status of the animals due to the loss of the entire fodder supply destinated for the winter period (Sedda, 2020).

In the early nineties, a fasciolosis outbreak has been observed on the basaltic Giara plateau in wild horses grazing in promiscuity with domestic ruminants, reservoir of *F. hepatica*. Following a particularly rainy season, a significant number of temporary water pools locally called ‘paulis’, and extended areas of shallow marshes were created, forming excellent proliferation conditions for *Limnea* (*Galba*) spp. In this situation, it was possible to detect fasciolosis also in these horses, an animal species in which fasciolosis does not represent a common parasitoses. This case illustrates how changed environmental conditions can increase metacercariae colonization of semi-disseminated pastures such as ‘paulis’, favouring the metacercaria ingestion by horses which are used to graze within these ‘paulis’ (Scala and Cancedda, 1993).

**Figure 1.**
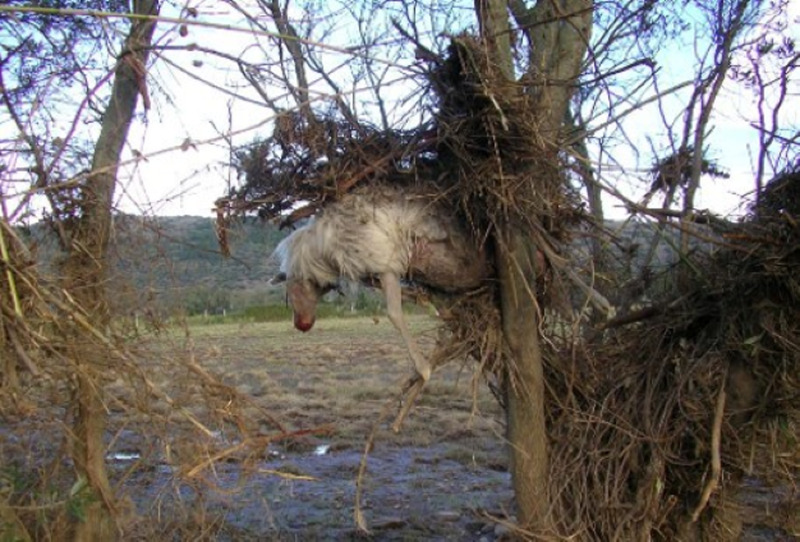
Sheep washed by the flood on a tree, north-eastern part of Sardinia Island (Italy), November 2013.

**Figure 2.**
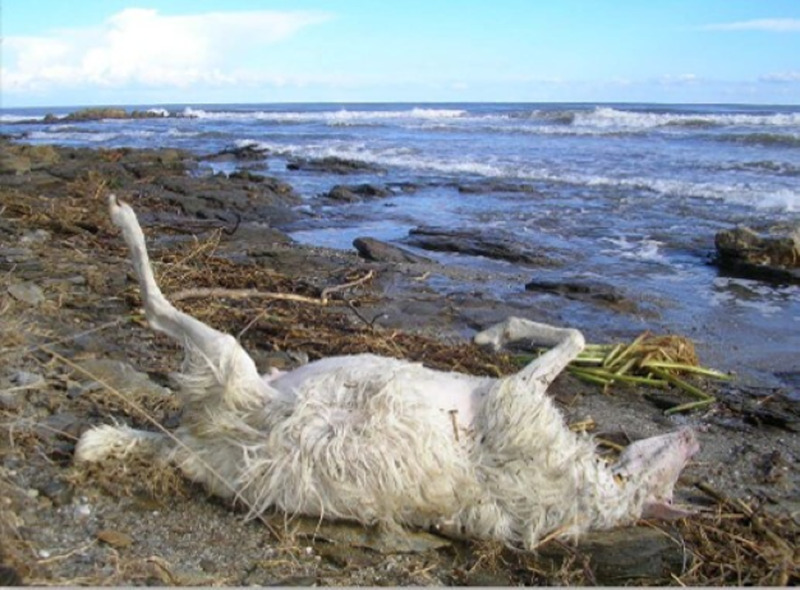
Sheep washed by the flood up to the sea, north-eastern part of Sardinia Island (Italy), November 2013.

Furthermore, the death of numerous husbandry animals reared in territories affected by such environmental catastrophes may also cause the geographical spread of metacestodosis, including those of zoonotic interest, such as cystic echinococcosis (CE). In fact, in Sardinia, during the floods of 2013, the death of a considerable number of sheep was documented, of which about 70% were estimated to be affected by CE (Scala *et al*., 2006). Their carcasses, abandoned on the pastures or carried away by flooding even over considerable distances (Figs 1 and 2) (in many cases up to the sea which then beached very far) were likely to get predated by feral and/or stray dogs and ingesting viscera possibly infected themselves with cestode species such as *Echinococcus granulosus*, *Taenia multiceps* and *T. hydatigena*.

### Other disastrous events possibly exacerbating flooding impacts on parasitosis

In the subchapters above it is illustrated how floods may lead to an increase in human and animal infections with parasitic agents. However, the fact that floodings, whether caused by heavy rains or by fluvial inundations involve also industrialized areas and thus may lead to environmental contamination with toxic chemicals or to the diffusion of contaminants already present in the soil, must be kept in mind. Such contaminations may affect large areas impacting directly on human and animal health but may also act on a wide range of organisms including free-living parasite stages.

Grigorieva and Livenets (2022) illustrate how floods in Russia can affect ecosystems and can lead to the release and diffusion of toxic chemicals and microbes already present in the soil. The authors estimated that there could be significant toxic and microbial health effects on people living near industrial or agricultural areas due to flooding.

In this regard, worth mentioning is the dioxin accident that occurred in Seveso (Milan province, Italy) in the 70ties. It was caused by the massive loss of the highly cancerogenic substance from the Icmesa plant, leading to the contamination of a large, densely populated area. This disaster frightened not only experts but the entire population of the affected areas and beyond. The *CBS Broadcasting Inc., an American commercial broadcast television and radio network,* has listed the disaster among the 12 worst environmental human catastrophes ever. Collecting earthworms in the contaminated area, it was possible to demonstrate that these worms contributed to moving dioxin from the surface into the soil. Of course, earthworms are not parasites, but excellent intermediated and/or paratenic hosts for many parasites (Martinucci *et al*., 1983). Unfortunately, to our knowledge, studies targeted to assess dioxin effects on parasites either free-living stages or present in contaminated hosts haven't been conducted.

In this context another chemical disaster that cannot be forgotten is related to pesticide production and happened in 1984, in India where a cloud of methyl isothiocyanate gas leaking from the Union Carbide pesticide plant killed about 10 000 people in Bhopal during the first three days. The disaster was blamed as the ‘genocide of the poor’. The tragic event prompted an animated debate among Italian parasitologists within the SoIPa, with colleagues sustaining the likelihood that this chemical soil pollution may impact on a wide range of organisms including parasitic forms present in pastures. Since the compound methyl isothiocyanate is a pesticide active on insect as well as nematode pests, its presence in the soil may modify the epidemiology of parasitic Diptera causing myasis and of gastro-intestinal nematodes species. Also in this case it would have been important to conduct studies on contamination effects not only in proximity of the plant but also in adjacent areas likely to be at secondary contamination risk because of specific hydro-geological characteristics.

## Fires: drastic immediate effects on parasites – complex long-term impacts on ecosystems

### Natural disaster fires *vs* deliberately set fires for parasite control

Considering the impact on ecosystems, fires consume not only the vegetation but also affect the upper soil layer where many parasites spend part of their life cycle, thus reducing habitat availability, with consequent potential benefits for the hosts (Álvarez-Ruiz *et al*., 2021). As reviewed by Scasta (2015), North American studies have illustrated how fires altered the micro-habitat of ticks and reduced the density of larvae, nymphs and adults (Scifres *et al*., 1988; Mather *et al*., 1993; Stafford *et al*., 1998; Cully, 1999).

In Tanzania and South Africa, similar reductions in ticks have been attributed to the drastic impact of fires on the micro-habitat. In this case fires were set deliberately due to concerns about tick-borne diseases such as babesiosis (Trollope, 2011). Although fire return intervals vary widely (from 3 years in tallgrass grasslands to 230 years in Douglas-fir forests) in African settings, fires are crucial in regulating ecosystems, ecological processes and pest dynamics (Agee, 1993; Anderson, 2006).

In Australia, (not to forget the massacre of koalas and marsupials in the 2021 bushfires) already in earlier fire events free-living stages of gastrointestinal parasites of sheep were found to be reduced (Hepworth and Hutchens, 2011) and going back to the 70ies, controlled burning of pastures has been recommended as a control measure to reduce gastrointestinal nematodes (GIN) (Barger, 1978). To light fires on pastures is also traditionally operated by Sardinian and Sicilian farmers at the end of the summer, with the intent to favour the revival of forage crops and to ‘sterilize’ the pastures from infesting parasitic forms.

Sheep examined in British Columbia, Canada, that had access to recently burnt areas had up to 10 times minor lungworm burdens (*Protostrongylus* spp.) than sheep kept on unburnt areas (Seip and Bunnell, 1985). In an area of the south-eastern United States bushfires were deliberately applied to destroy the micro-habitat of the gastropod hosts of *Parelaphostrongylus tenuis,* the brain meningeal worm. This intervention allowed to control the parasites’ diffusion, proven to have devastating effects on native ungulates such as elk and deer (Weir, 2009).

### Impact on sheep breeding and risks of parasite diffusion in Sardinia

**Figure 3.**
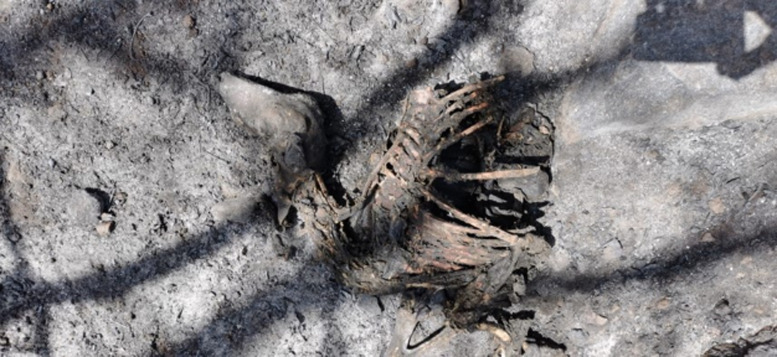
Carbonized sheep in the area of Montiferru (central-western area of Sardinia), after the fire in summer 2021.

In Sardinia, concerns were raised that the death of numerous animals killed by fires may increase the diffusion of cestode species, as in the case of floods. However, this most probably did not occur in the areas affected by the 2021 fires, since the carcasses of the sheep and other animals caught by the fires in the pastures or in their shelters were charred (Fig. 3). Furthermore, on-the-spot inspections carried out in the affected areas for more than 30 days after the event did not reveal the presence of feral and/or stray dogs.

The epidemiology of some parasitoses most likely was also influenced by the transfer of sheep from original burnt grazing areas to other pastures with different pedoclimatic characteristics in which ‘new’ or ‘different’ parasite forms or agents of the herd pathocenosis may have been present. Such newly encountered parasites such as *Dicrocoelium dendriticum*, *F. hepatica* or different species of bronchopulmonary and/or gastro-intestinal nematodes, may have established themselves in the transferred animals, which could then, in the absence of adequate control measures, have been imported into the original grazing areas with the return of the animals on their home pastures. Obviously, such areas, at least in the first season of reintroduction of grazing animals, would be ‘sterile’ regarding L3 contamination of GIN, possible cestode eggs and the presence of intermediate hosts.

Unfortunately, these fire disasters have caught many public services in Sardinia unprepared to tackle adequately the numerous husbandry/sanitary problems that have arisen. On many occasions, not even the actual number of animals involved was available. From the Sardinian experiences emerge the need for solid, area specific data on the ‘normal’ epidemiology of endoparasitoses in grazing animals but also for assessments of parasitological risks according to type of disaster. Such knowledge may help guiding prevention and mitigation programs at farm and community level by local services.

Regarding the increase in frequency and extension of fire disasters in Sardinia, this may be related also to the depopulation of rural areas and their consequent re-naturalization. Abandonment of agricultural activities brought the proportion of forest over the total area to grow from 13% in the early 50ties (Camarda and Vacca, 2021) to the current 54%. Over the last decade, population loss has become an alarming phenomenon all over Italy mainly affecting small municipalities in half of the 20 regions, from North to South, with a negative population trend observed in over 50% of the area in the affected regions (Amodio, 2022).

Therefore, to contain fires and mitigate their effects, it is most important to adopt at farm level preventive measures such as: improve the structure of fences including escape routes for animals; secure sheds (primarily barns); create water points; provide viability in every direction and part of the farm; provide food supply for animals (Camarda and Vacca, 2021). In addition, the promotion of silvopastoralism as a wildfire prevention strategy should be considered (Nuss-Girona *et al*., 2022). According to the local forest characteristics and type of breeding practiced in the area, various ruminant species might be employed (Nuss-Girona *et al*., 2022). In this context, also the grazing of horses has been found effective in controlling easily combustible underwood vegetation in an area of Spain (Rigueiro *et al*., 2002). Other Iberian experiences demonstrate how the breeding of donkeys, goats and sheep, the latter together in large flocks, are able to reduce the risk of fires compared to areas where abandonment has induced natural reforestation. (Bartolomè *et al*., 2020). Botanical studies have allowed to identify the plant species involved in the phenomenon (Lasanta-Martínez *et al*., 2005; Mancilla-Leytón *et al*., 2013). In Italy, however, experiences in this sector are lacking and mainly directed to the organoleptic characteristics of animal products from extensive husbandry (Pugliese *et al*., 2006).

## Droughts: slowly killing disasters – time remaining to react

### Impact on GIN transmission and evasive grazing control strategies

Regarding GIN, the dynamics of larval development in response to climatic variables such as temperature and humidity has been studied extensively, both in the laboratory and the field, and various models have been developed to predict infection risks of ruminant hosts (Vineer *et al*., 2020). With the help of such models the design of GIN control programs is facilitated, allowing to reduce pharmacological treatments to a minimum and intervene with alternative measures such as evasive grazing. However, the predictive capacity of such models might be challenged by exceptional events. Indeed, extraordinary heavy rains, extremely high temperatures or prolonged drought periods may create conditions that possibly exceed value ranges of the climatic parameters considered in the models.

A drought period that struck the Netherland in summer 2003, allowed Eysker *et al*. (2005) to observe the capacity of *Haemonchus contortus* to adapt to environmental stress: whereas the development of free-living stages was poor as expected, L3 larvae that have matured before the drought period reached to survive in comparable numbers as in previous years. Moreover, in the absence of re-infection, adult nematodes were found to survive in the hosts for up to 3 months (Eysker *et al*., 2005). This example illustrates the complexity of parasite–host–environment relationships, but also suggests that programmed parasite control will be more and more difficult to realize in the era of increasing extreme events. Evasive grazing programs for GIN control will have to cope not only with altered parasite transmission patterns, but also with changed pasture availability. In an area affected by drought, grazable plots will be reduced in numbers and the quality of the forage altered, possibly affecting the nutritional status and resilience of ruminant hosts. Until now, evasive grazing as a GIN control strategy has been considered indispensable, since it allows to reduce numbers of anthelmintic treatments and thus contributes to delay resistance development of GIN species to available drugs. Drug and multiple drug resistance has already become a serious threat in many contexts, but the expected increase in frequency and intensity of droughts may even enhance the problem, given that drought conditions decrease the amount of free-living GIN stages, i.e. reduce the proportion of the nematode populations in *refugia* (Knapp-Lawitzke *et al*., 2016).

Experiments were performed to assess the effects of drought stress and different temperature/humidity ranges over time on the survival and fitness of *Cooperia oncophora* L3 and their distribution in grass and soil under controlled conditions using a climate chamber (Knapp-Lawitzke *et al*., 2016). Weed containers inoculated with L3 were analysed after 1–6 weeks using descriptive statistics and linear modelling. A large proportion of L3 was recovered from the ground where fitness was even better preserved than on grass. The obtained results highlight the relative importance of these factors and will help to design better models for L3 population dynamics on pasture in the future. Furthermore, the results of these investigations may offer explanations concerning the interdependencies of the development of anthelmintic resistance and the presence of hot/dry climatic conditions (Knapp-Lawitzke *et al*., 2016)

In contrast to floodings or fires, droughts are not causing situations of immediate emergency. Farmers and breeders are given the time to frame the problem and intervene to mitigate effects on husbandry production parameters and to prevent levels of parasite infections that may lead to disease (Morgan and Wall, 2009). However, to tackle these new challenges, breeders should be assisted more closely by veterinary practitioners and breeders’ associations. As an example, during 2021, the Marche Region (Central Italy) experienced an exceptionally dry summer that forced sheep breeders in the Apennine Mountains to modify grazing practices and integrate feeds with hay. Thanks to regular monitoring of gastro-intestinal parasites conducted in local sheep farms by staff of the University of Camerino (Marche Region), a substantial increase of GIN egg counts in autumn was detected in some of the monitored farms. Prompt anthelmintic treatment of the sheep groups involved, allowed to preserve the good health status of the animals. This example suggests that intensified parasitological monitoring should become the first line response to climate change. Presumably, more frequent parasitological examinations increase production costs, however regular monitoring might still prevent more substantial expenses linked to parasite born disease outbreaks.

### Impact on the geographical distribution of parasite species

Another example of extreme climatic events’ impact on parasites, comes from the recent dramatic drought episodes in Sicily in 2017: in that occasion emerged how such events may affect the geographical distribution patterns of parasites, in this case of ectoparasites that seemed to belong to the distant past, namely leeches; once used for therapeutic purposes (hypertension, bloodletting, swelling) due to their ravenous hematophagy or employed for cosmetic purposes for colouring black hair, beard and moustache (Hoeppli, 1959). During the heavy drought episode, leeches of the species *Limnatis nilotica* were found to have established in high densities in remnant water puddles, provoking significant lesions in the oral cavity of the animals that took water from those puddles. In the absence of *Hirudo medicinalis* this represented the first observation of the species *L. nilotica* in an Italian region (Arfuso *et al*., 2019).

## Geographic information systems (GIS) – bringing complexity under control

The risks related to climate change are delineating new geographies that can be called ‘risk geographies’ whose borders are not traditional administrative boundaries, but rather are traced by the dangers and vulnerabilities of areas and the different capacity of animals and communities to react to new climatic conditions and to emergencies (Galderisi *et al*., 2020). For example, after the earthquake in Emilia-Romagna region during 2012, Modena colleagues prepared a specific emergency operating manual for ‘seismic geographies’ in Italy with which to equip Municipalities responsible for the implementation of rescue activities through the COCs (Municipal Operational Center). The operating manual contains addresses, telephone numbers, availability of: earth moving machines, trucks for transporting animals and carcasses, slaughterhouses, breeding units, processing industries and any other information that may be useful in emergency situations (Ferraresi *et al*., 2016). The geo-referenced manual allowed to appreciate the utility of GIS applications for an integrated management of animal health within an emergency context in a One Health perspective.

In this context, GIS associated to new electronic devices [e.g. GPS (Global Positioning System), drones, microclimate data loggers], can provide essential support in the assessment of climate change impact on parasites transmission. For several years, the epidemiological maps complemented by univariate and multivariate statistical analysis have been used in veterinary parasitology for disease mapping, ecological analysis and epidemiological surveillance, becoming indispensable tools for processing, analysing and visualizing spatial data (Rinaldi *et al*., 2006). More specifically, GIS can be used for the evaluation of the spatial distribution of parasitoses, the identification of statistically significant clusters of infection, the determination of the main environmental conditions favouring the parasites transmission and the prediction of the areas with highest risk of infection. Data collected at farm level would allow veterinary practitioners to implement targeted treatment schemes, i.e. employ pharmacological treatments only when needed and only for groups of animals with relevant parasite burdens. Thus, farm scale GIS information may allow for a more attentive use of drugs which is key to slow down emergence and diffusion of drug resistant parasite populations.

A thorough knowledge of the microepidemiology of a territory can be extremely useful to manage the effects of natural disasters related to extreme weather conditions, especially in a country like Italy, one of the most vulnerable territories in the Mediterranean area. GIS applications have the potential to generate constantly updated maps providing a detailed micro-epidemiological status of an area at small or large scale. In this perspective, the collection of high spatial-temporal resolution data (e.g. occurrence of infection, microclimatic conditions, farm management practices), is necessary but at the same time a complex task.

An effective support is offered by innovative strategies for parasites control, based on the combined use of GIS, electronic devices and other technologies which allow the monitoring of animal health in a continuous and automated way, and can be exploited for the improvement of the productivity and the detection of health issues at an early stage (Schillings *et al*., 2021). The combined use of GIS software and electronic wearable devices such as collars, that replace the classic cowbell with a modern instrument such as GPS, provides real-time monitoring of the movements of animals and thus allows to circumscribe the areas where they spent most of their time (grazing patterns). Unmanned aerial vehicles (drones), already used to capture habitats (e.g. small water bodies) of parasites and vectors at very small scales (Charlier *et al*., 2014), have been recently used to facilitate the treatment of animals in remote grazing areas with the release of medicated baits (Yu *et al*., 2017). The installation of micro-climate data loggers for the monitoring of soil surface temperature and humidity in grazing areas, offers the possibility to identify the environmental conditions which favour the local abundance of intermediate hosts for snail-born parasitoses.

To identify areas at risk of parasitoses outbreaks as a consequence of extreme weather events, predictive models can be developed using GIS combined with Artificial Intelligence (AI) programs. The machine learning algorithms allow the identification of environmental and meteorological variables that influence the spread of parasitoses in the environment. In the first training step, the algorithm is used to identify relationships between explanatory variables and the predicting variable (presence/absence of a given parasite). In a second step, the model predicts the risk of infection in the study area and in other locations characterized by the same environmental explanatory variables (Bosco *et al*., 2021).

## Concluding remarks

Climate change with its devastating consequences calls for concerted actions at global level to stop global warming. However, actions to prevent natural disasters or at least mitigate their effects must and can be taken at various levels, from the single farmer to the municipality, to the regional level; all stakeholders covering every relevant discipline must be involved in a One Health approach. Such actions inescapably need to be based on knowledge, calling researchers to recognize their responsibilities as public ‘knowledge workers’ and to invest themselves in the climate change challenge. Cutting edge methodologies and technological innovations developed by the various scientific disciplines must be fully exploited.

Extreme weather events pose a risk to public health all over the world in both industrialized and developing countries, but the impact will be disproportionately hitting the latter and thus exacerbate the already existing health disparities (Cann *et al*., 2013).

As illustrated above, GIS applications combined with AI programs are examples of novel, formidable tools that allow the collection of spatial/temporal data at whatever level desired. Concerning risk assessment for epidemics caused by parasitic agents, the examples cited above demonstrate the usefulness of GIS applications, suggesting their wider employment in a more systematic, prevention-oriented manner. For example, GIS elaboration of areas at high risk of inundations combined with the geo-referenced information on cattle farms and *Cryptosporidium (*zoonotic genotypes*)* diffusion on those farms, would allow to advice veterinary practitioners to implement strict anti-parasitic control measures on such farms, and thus minimize the risk of human infection due to oocysts contamination of water resources after floodings. Similarly, GIS and AI programs will help to design better models for other water borne protozoans parasites, but also for *F. hepatica*, GIN species, arthropod vectors and vector-borne diseases. In general, knowing *a priori* the territory and the epidemiology of parasites using GIS is key for the design of effective control strategies able to increase the resilience of animals and humans to parasitic infections, and their ability to recover from natural hazards related to climate change.

Fire ecology and parasitology should be considered priority areas for future research, sustaining that interdisciplinary collaboration between specialists of fire ecology, parasitology, animal production and human health can have far-reaching benefits for society if tackled by a One Health approach (Scasta, 2015).

Regarding actions to be taken for tackling the various climate change issues, there is an obvious difficulty to recognize fundamental and technological knowledge already available and to put it into practice for the scope. In this respect also the parasitologists community must improve performance and act promptly. Beyond publishing research results within the academic environment more emphasis should be put on conveying the acquired knowledge to health professionals such as medical doctors and veterinary practitioners, health professionals’ associations, health authorities at community, district and at national level. An informed veterinary practitioner would thus be capable to effect overall and parasitic infection specific risk assessments on farms and elaborate setting tailored preventive measures as well as adequate emergency interventions in case of disasters. Similarly, an informed community, knowing to be situated in an area at high risk for flooding, would for example, set up a mobile phone alert facility to promptly inform the population in case of event and make sure in advance that water purification plants are equipped with high-capacity filter systems to prevent possible *Cryptosporidium, Toxoplasma* or other infectious agents’ contamination of drinking water (Karanis *et al*., 2007).

The symposium at the National Congress of the SoIPa was a first occasion for Italian parasitologists to reason about ‘disaster parasitology’ and researchers’ responsibilities, in tune with the ‘Carta di Rieti’ (Chart of Rieti) which calls for responsible communication in the event of natural disasters (Pompili *et al*., 2018). Thoughts and discussion shared during the aforementioned congress and presented in this study might guide priorities for disaster parasitology at broader European and global scale.

**Figure 4.**
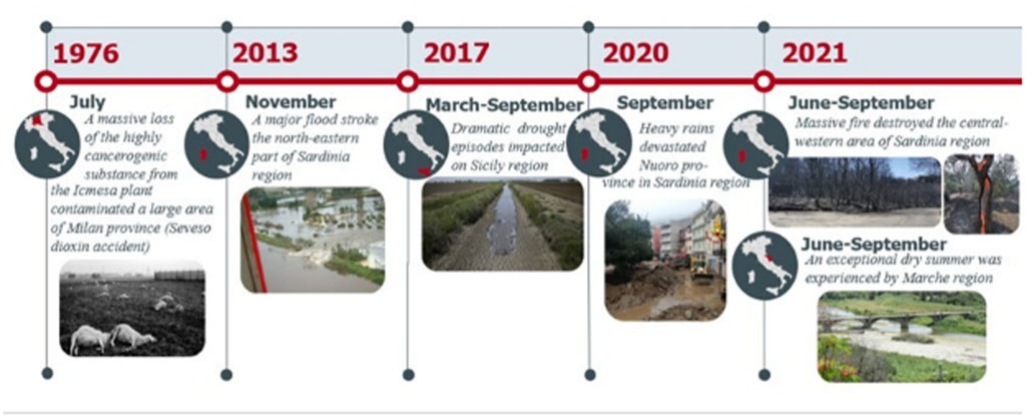
The timeline of Italian disaster examples, affecting the parasite environmental habitats, their survival and capacity to propagate.

Many of the considerations forwarded in this article are drawn from direct experiences of the authors involved in recent disasters caused by floods, fires and droughts in various parts of Italy (resumed in Fig. 4). Having been involved in those events as parasitologists in various roles, the authors care to propose 6 take home messages to the parasitologist community for further reflection (Box 1).

Box 1. Take home messages:

- Advocate the recognition of disaster parasitology as *a priori*ty research area by the entire parasitologists community: prompt research endeavours are urgently needed and issues to be tackled in an interdisciplinary and multisectoral One Health approach.
- Recommend regular parasite monitoring for the implementation of rational parasite control at farm level and for elaborating parasitological maps to predict disease risks for animals and humans in areas likely to be flooded or vulnerable to other extreme weather events.
- Promote extensive animal husbandry practices to contrast abandonment of marginal rural areas and encourage silvopastorism with equines and small ruminants as a wildfire prevention measure.
- Stimulate the use of novel research tools such as GIS applications and AI programs for the sake of increasing disaster parasitology understanding, but also – importantly – to guide prevention, preparedness and emergency planning in ‘geographical’ risk areas.
- Advocate thorough dissemination of research results to governmental institutions, decision makers and professionals at all levels. In the era of climate change, dissemination of knowledge must be endorsed by the parasitologists community as a mission.

Take action by elaborating and providing guidelines and educational information tools on disaster prevention and preparedness to all stakeholders, such as emergency guidelines for health professionals, awareness raising lectures for students, disaster specific prevention courses for breeders and farmers.

## Data Availability

Not applicable.
